# Thoracic Aortic Aneurysm Surgery: DON'T QUIT—JUST DO IT

**DOI:** 10.1055/s-0042-1750098

**Published:** 2022-12-15

**Authors:** Vicente Orozco-Sevilla, Ginger Etheridge, Joseph S. Coselli

**Affiliations:** 1 Division of Cardiothoracic Surgery, Michael E. DeBakey Department of Surgery, Baylor College of Medicine, Houston, Texas; 2 Department of Cardiovascular Surgery, Texas Heart Institute, Houston, Texas; 3 Department of Cardiovascular Surgery, CHI St. Luke's Health—Baylor St. Luke's Medical Center, Houston, Texas

**Keywords:** aortic surgery, arch aneurysm, thoracoabdominal aneurysm surgery

## Abstract

Surgical aortic repair has progressed from aneurysm ligation to homografts to Dacron grafts to totally endovascular interventions. These fields will continue to evolve, and new endovascular technology will be used in virtually every part of the aorta, eventually dominating this field of surgery. However, as surgeons, we must be cautious and not let go of our open-surgery skills, as they will always be the ultimate bailout solution.

## Introduction

Aortic surgery has undergone a steady and innovative evolution—rather than a revolution—since the 1950s, resulting in remarkable changes. Pioneers in the field include Drs. Michael E. DeBakey, Denton A. Cooley, and E. Stanley Crawford, who laid the foundation for modern aortic surgery. In their heyday, cardiovascular surgery was vastly different from what we know today, and it would be fascinating to hear the perspective of these legendary surgeons on current open, hybrid, and endovascular innovations.

Before 1950, attempts to repair aortic aneurysms were largely ineffective 1 ; these approaches included cellophane wrapping, proximal ligation, and wire coiling to try to thrombose the aneurysm. The introduction of aortography by dos Santos of Portugal significantly contributed to the understanding and advancement of the field. By the 1950s, Beall, Seldinger, and other clinicians were able to improve the accuracy and safety of translumbar aortography, reducing the frequency of complications. 2

Crawford's iconic *Diseases of the Aorta: An Atlas of Angiographic Pathology and Surgical Treatment* contains illustrations of hundreds of cases of aortic pathology and aortic surgery. Preoperative aortography was used to plan the procedures before the advent of computed tomography (CT) scans and other advanced imaging techniques. During the early days of DeBakey's groundbreaking work, cross-table lateral radiographs were used to detect calcium in the infrarenal abdominal aorta, followed by an intravenous pyelogram to search for displacement of the ureters. In 1976, Axelbaum et al 3 performed the first CT imaging study to evaluate aortic pathology, and since then, this modality has become the preferred method of diagnosis and preoperative planning to treat aortic aneurysm. The ability to detect disease by using CT was a monumental advance; accordingly, Sir Allan Cormack and Godfrey Hounsfield were awarded the Nobel Prize in 1979. CT scans are now widely available and have dramatically changed the way we treat our patients. As a result, asymptomatic aortic aneurysms, especially thoracic aortic aneurysms, are diagnosed and treated at a faster pace.

Unlike surgery to treat aortic coarctation, which relied on simply transecting the aorta and suturing it back together, a different approach had to be developed to replace the aorta and restore its continuity: namely, the resection and replacement of the aortic tissue with a graft substitute. The ideal prosthetic aortic replacement graft would be long-lasting, nonthrombogenic, infection-free, and biocompatible. Homografts obtained from cadavers were tried first, being used in the early work of nearly all midcentury aortic surgeons, including Crawford, Cooley, and DeBakey. In the early 1950s, Voorhees' work developing synthetic grafts made of Vinyon-N inspired DeBakey to develop the first aortic replacement grafts made from Dacron, 4 which is still the dominant synthetic material used today. The initial Dacron material was porous, which caused excessive bleeding in heparinized patients. In early efforts to address this problem, the graft was soaked in nonheparinized blood that was drawn from the patient. Later, other techniques were used to seal the graft's pores, including soaking the graft in plasma or albumin and then autoclaving it for 5 minutes. This technique was popularized by Crawford, Livesay, and others. 5 Today, grafts are sealed with collagen, albumin, and gelatin, among other materials.

**Fig. 1:**
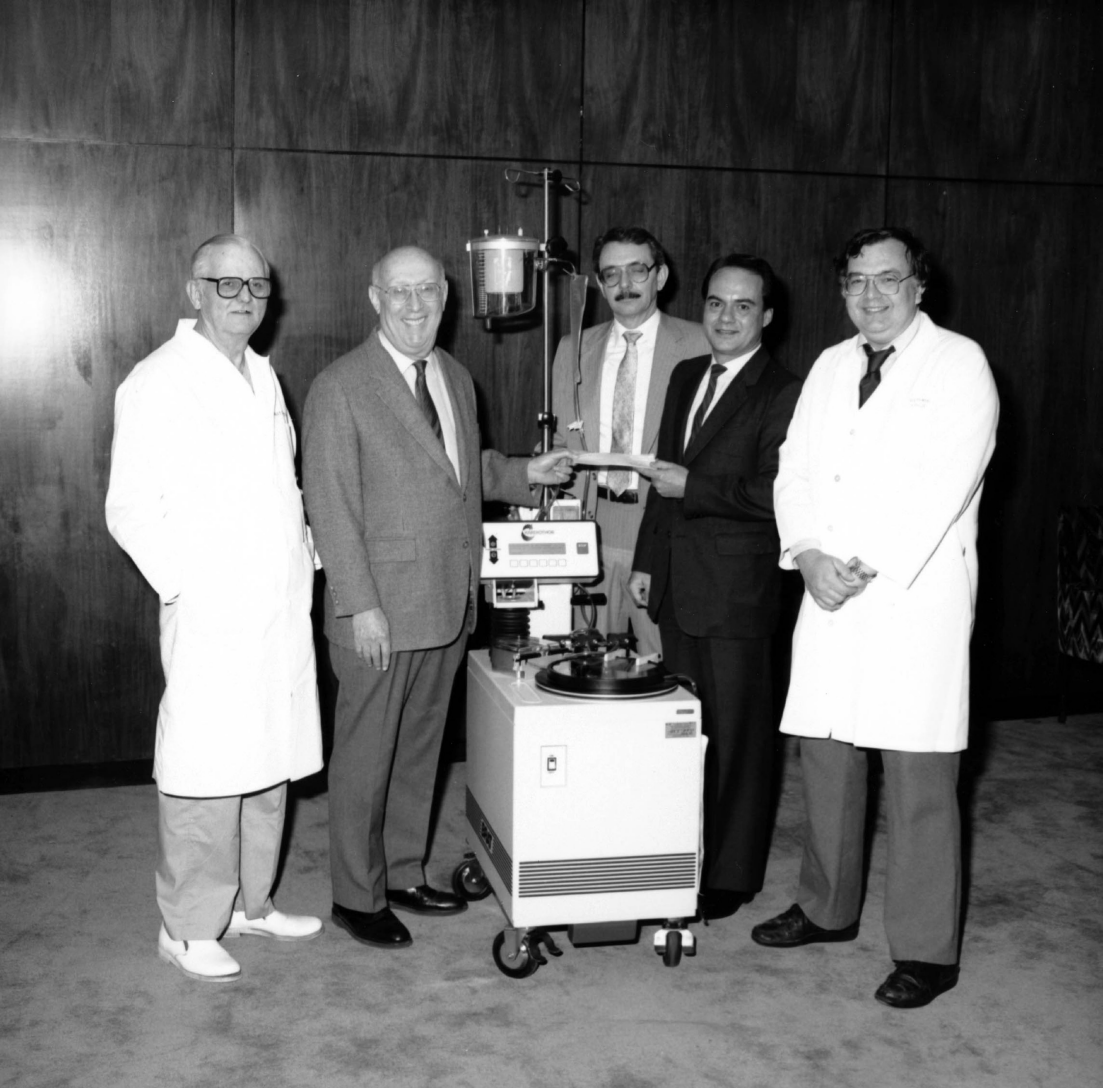
Drs. Crawford and Yawn and Lou Feldman (from Baylor College of Medicine's machine shop) with the Baylor Rapid Autotransfusion (BRAT) cell-saver machine in 1988. Used with permission from Baylor College of Medicine.

Blood transfusions and quantity of shed blood were a problem during extensive aortic repair. Yawn, Crawford, and Feldman collaborated on the development of the Baylor Rapid Autotransfusion (BRAT) cell-saving device ( Fig. 1 ), which collects shed blood, spins it down to wash it, and returns it to the patient, thereby reducing the need for blood transfusions. Although others previously developed similar systems to return shed blood, the BRAT device was efficient and relatively easy to use.

## Aortic Arch

**Fig. 2:**
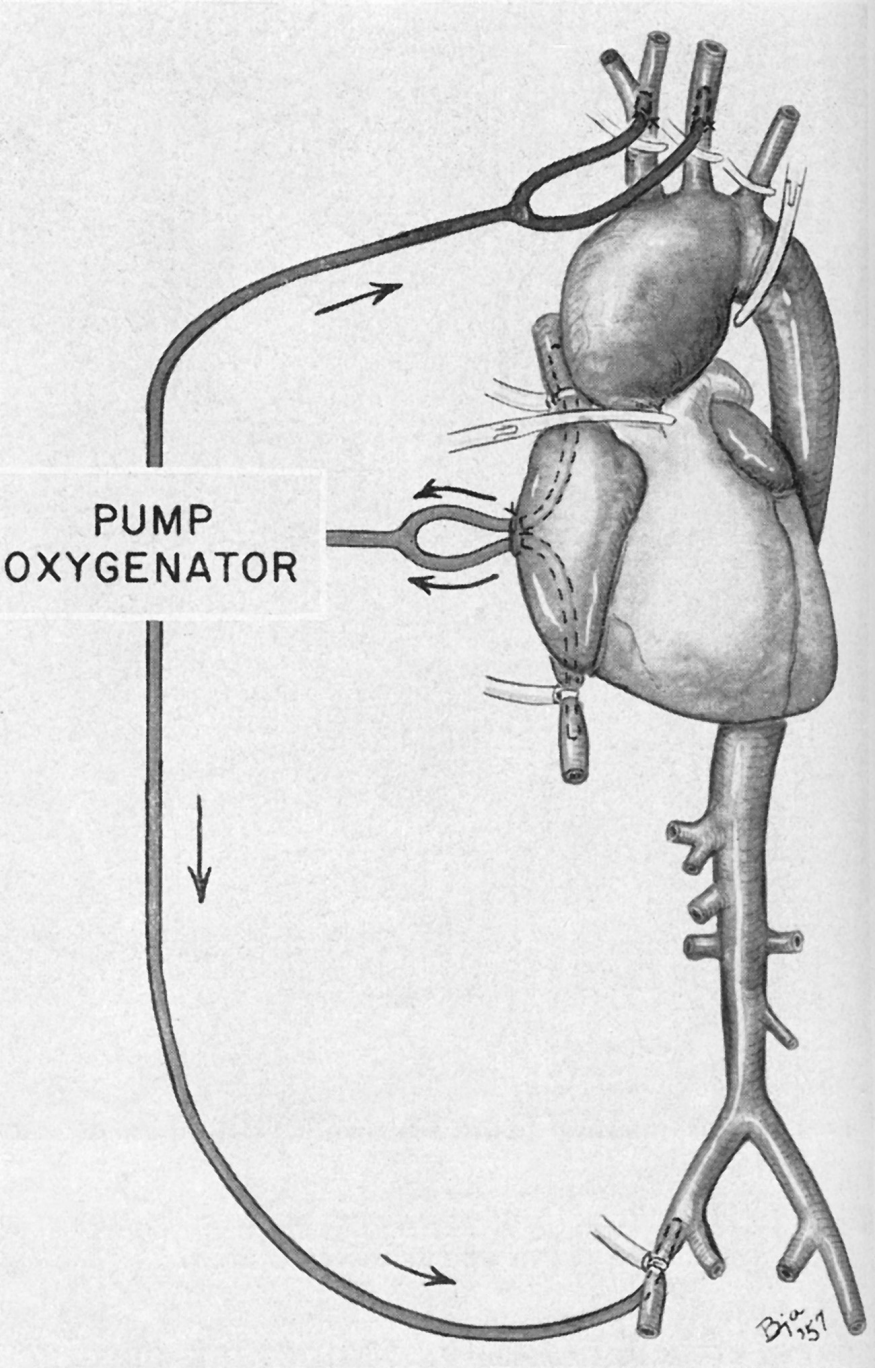
The first successful attempt by DeBakey et al to replace the aortic arch with a homograft (1957). Cardiopulmonary bypass was used to facilitate an early form of antegrade cerebral perfusion. Used with permission of Baylor College of Medicine.

In 1957, DeBakey et al 6 were the first to successfully treat an aortic arch aneurysm ( Fig. 2 ). While cardiopulmonary bypass (CPB) with bilateral antegrade perfusion via the brachiocephalic vessels perfused the brain, the transverse aortic arch was resected and replaced with a homograft. Notably, their method of cerebral protection is similar to that used in contemporary repair, although hypothermic protection is now additionally provided.

It is critical to safeguard the brain during aortic repair involving the disruption of brachiocephalic vessels. In the early 1950s, Bigelow et al 7 in Toronto pioneered total body cooling with ice baths and cooling blankets. The area of brain protection was advanced by the work of Griepp et al 8 in 1975; they introduced the use of hypothermic circulatory arrest (HCA) using heat exchangers incorporated into CPB circuits, in which patients were simply cooled (with no additional cerebral protection) to a low temperature of 14°C, allowing reconstruction of the aortic arch. Using HCA provided good conditions for inspecting the arch because the heart was arrested, temporarily resulting in a blood-free field. The patient was then carefully rewarmed with CPB. Griepp et al's seminal report described four patients who underwent complete aortic arch replacement, three of whom survived.

For the next four decades, Griepp's approach was the mainstay of total aortic arch replacement and came to be known as deep HCA. However, the limits of this approach—specifically, how long the heart could be safely arrested—were unknown. Svensson et al 9 reported on their experience with deep HCA in 656 patients undergoing total arch replacement from 1979 to 1991. If the period of circulatory arrest exceeded 40 minutes, complications increased dramatically; the stroke rate in these cases was reported to be 7%, and operative mortality was 10%. Further supporting the need for expeditious repair, studies conducted by McCullough et al 10 showed that even at extremely low temperatures (10–15°C), metabolic activity within the brain is highly active and continues to use substantial amounts of oxygen and glucose.

Since the use of deep HCA alone became a widely used approach, there has been a worldwide search for ways to improve brain protection for complex aortic arch cases that push the limits of deep HCA. One solution was to use retrograde cerebral perfusion via the superior vena cava, which was first used to protect against embolism by flushing the operative field and was later popularized by Ueda et al 11 in 1988 as a method of brain protection during arch repair.

Another solution was to revisit the use of antegrade cerebral perfusion (ACP). Although DeBakey had used ACP in the 1950s, this approach was not widely adopted because of the technological limitations of the equipment available at the time. The use of ACP was reinstituted by Kazui et al 12 and Bachet et al 13 in the early 1990s with improved equipment, and since then, ACP has become the dominant technique of providing cerebral perfusion during arch repair.

Important data such as those provided by a meta-analysis of ACP studies by Tian et al 14 allowed us to move from extremely cold temperatures of 18 to 20°C to much warmer temperatures (26.5°C) with no increase in mortality, lower rates of stroke and renal failure, and a significant reduction in time spent cooling and rewarming the patient, as well as fewer problems with hypothermia and coagulation.

The arterial cannulation sites used to provide ACP have changed over time. Initially, femoral cannulation was used in almost every case, until Sabik et al 15 proposed the use of axillary artery cannulation. This has been particularly valuable in operations for aortic dissection. Depending on the pathology, we now use axillary, innominate, subclavian, carotid, femoral, transapical, transseptal, or direct aortic cannulation.

In time, techniques were employed to tackle complex and extensive aneurysms involving both the ascending aorta and aortic arch, as well as the descending thoracic or thoracoabdominal aorta. Borst et al's elephant trunk technique 16 facilitated a two-stage repair by providing an additional piece of graft material (i.e., the trunk) to create a secure graft-to-graft anastomosis. Today, along with the first stage of the elephant trunk repair, there are many different types of arch repair, including island repair, debranching or Y-graft techniques, and combinations of both. The elephant trunk technique has evolved to include thoracic endovascular aortic repair (TEVAR) techniques, namely the “frozen elephant trunk” repair, which is named so because the extension is more rigid than the classic elephant trunk approach. In 1996, Kato et al 17 introduced the frozen elephant trunk concept.

## Descending Thoracic Aorta

DeBakey and Cooley 18 are credited with performing the first successful descending thoracic aortic (DTA) aneurysm repair in 1953. The fully resected aneurysm was replaced by a homograft in an open repair via a thoracotomy and with proximal aortic cross-clamping. At that time, cross-clamp time was usually only the 10 to 12 minutes needed for suturing two anastomoses as part of a simple “tube” graft replacement. Open DTA replacement was a reliable operation with excellent results. In some cases, left heart bypass (LHB) was used to protect the kidneys and perfuse the body distally while some or all of the repair was performed. In 1993, Svensson et al's 19 study of 832 patients who underwent open repair of DTA aneurysm showed that significant progress had been made in the technique, reducing the mortality rate from 15% in 1956 to 1982 to 2% in the early 1990s. Looking at our own institutional experience, we examined the results of 428 DTA aneurysm repairs in 2004. 20 After excluding 41 patients who required HCA, we compared the results between 45 patients who had LHB and 340 who did not. The frequency of paraplegia/paraparesis was low and did not differ between DTA repair patients with and without LHB; thus, we felt that a “clamp-and-sew” repair was sufficient for DTA aneurysm repair.

In the treatment of DTA aneurysm, TEVAR has largely replaced open repair. Endovascular stent-grafts for the DTA are widely available around the world and have become the treatment of choice for DTA aneurysm. However, open repair remains the gold standard for treating certain cases of post-TEVAR aneurysm expansion, for treating patients with heritable connective tissue disorders, and for treating patients with infection of the aorta.

## Thoracoabdominal Aorta

Etheredge et al 21 performed one of the first thoracoabdominal aorta repairs in 1955, using a homograft to replace the aorta, reattaching the celiac and superior mesenteric arteries while sacrificing the left kidney and preserving the right renal artery and using a temporary shunt to provide distal aortic perfusion during repair. Soon thereafter, DeBakey et al 22 treated four patients with a similar approach that relied on the use of homograft replacement. In three of the four patients, temporary shunts were used to aid distal perfusion, and in the one patient, mild hypothermia was used. Two of the four patients survived.

**Fig. 3:**
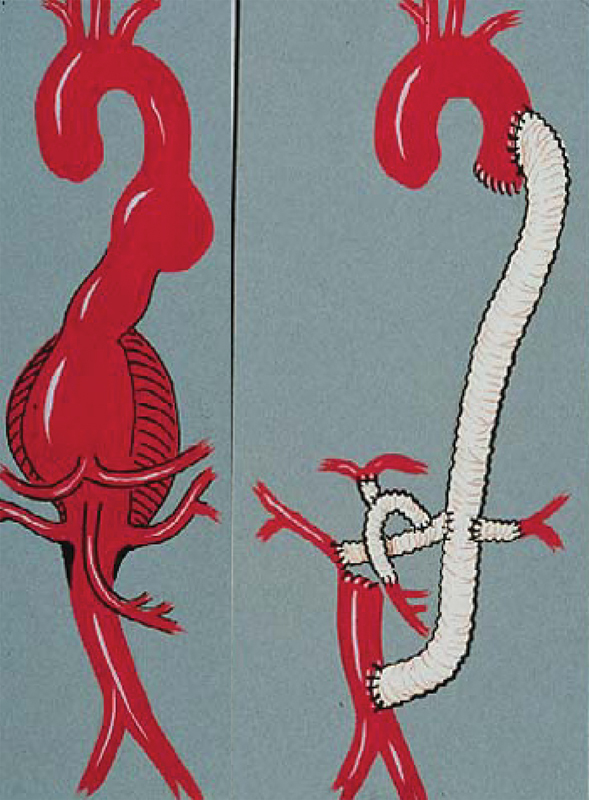
DeBakey's extra-anatomical approach to thoracoabdominal aortic aneurysm repair relied on anastomosing a Dacron graft around the aneurysm before reattaching the visceral arteries with bypass grafts and subsequently extirpating the aneurysm. Used with permission of Baylor College of Medicine.

These advancements were further detailed in a report by DeBakey et al 23 in the 1960s, which described 42 cases with a 26% ( *n* = 11) mortality rate. Here, an entirely different method was used to replace the aorta—DeBakey's extra-anatomical approach. Relying on the use of synthetic grafts (i.e., grafts made from Dacron and other synthetic fabrics), the extra-anatomic approach used the graft as an initial bypass graft that was placed around the aneurysm and anastomosed at the top and bottom. The aneurysm was then extirpated, and the visceral arteries were reattached with individual branch grafts ( Fig. 3 ). Among this study's many findings was that paraplegia remained a predominant problem in patients who underwent thoracoabdominal aortic aneurysm (TAAA) repair.

**Fig. 4:**
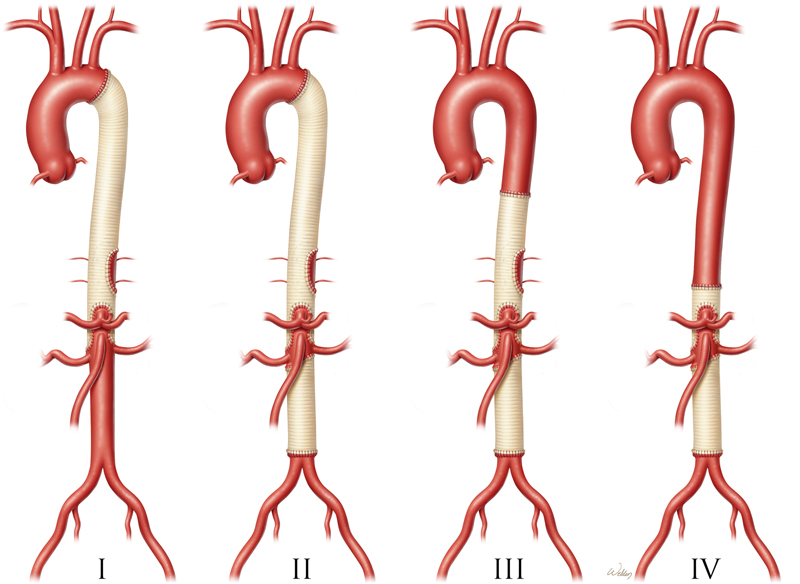
The classification E. Stanley Crawford used to describe thoracoabdominal aortic aneurysm repairs I to IV according to the extent of the aorta replaced, with extent II repair being the most extensive. Used with permission of Baylor College of Medicine.

Crawford, a colleague of DeBakey for many years, is widely regarded as the godfather of thoracoabdominal aneurysm surgery. He changed the method of the operation from DeBakey's extra-anatomic bypass approach to an intra-aneurysmal reconstruction that followed the anatomic position of the aorta, as well as the use of island patches to reincorporate branching arteries into the repair. 24 Thus, the evolution of thoracoabdominal repair had moved from the homograft replacement (1950s) to DeBakey's extra-anatomic bypass (1960s) to a more anatomic approach with Crawford (1970s). As a result of this advancement, Crawford 24 was able to reduce mortality to 8%, as reported in his landmark paper. Later, Crawford described a method to categorize the extent of aortic repair by using Crawford extents I through IV ( Fig. 4 ), with extent II being the most extensive repair of the distal aorta. According to Svensson et al's 1993 report, 25 Crawford's lifetime experience of 1,509 thoracoabdominal aortic repairs performed between 1960 and 1991 resulted in an early mortality rate of 8% ( *n* = 123) and a paraplegia rate of 16% ( *n* = 234). Thus, paraplegia continued to be a problem in patients who underwent extent II repairs.

Although the spirit of Crawford's approach to TAAA remains, we have refined our techniques over time. Our group did not use heparin in Crawford's day, but we do now. We have also transitioned from the clamp-and-sew technique to the selective use of LHB, cerebrospinal fluid (CSF) drainage, and visceral perfusion, as well as cold renal perfusion whenever possible. Additionally, we have moved from the island technique to the selective use of branched grafts to minimize residual aortic tissue in the “visceral patch” and prevent late patch aneurysms; this approach includes the use of a four-branched thoracoabdominal aortic graft of our own design. Our randomized trials show that cold renal perfusion and CSF drainage have benefits, and a retrospective study established the benefits of LHB. Our randomized trial exploring the use of CSF drainage in extent I and II TAAA repairs, published in 2002, enrolled 156 patients between 1997 and 1999. 26 Results suggest that the use of CSF drainage in such repairs reduced the incidence of paraplegia from 13.0 to 2.6% ( *p* = 0.03).

**Fig. 5:**
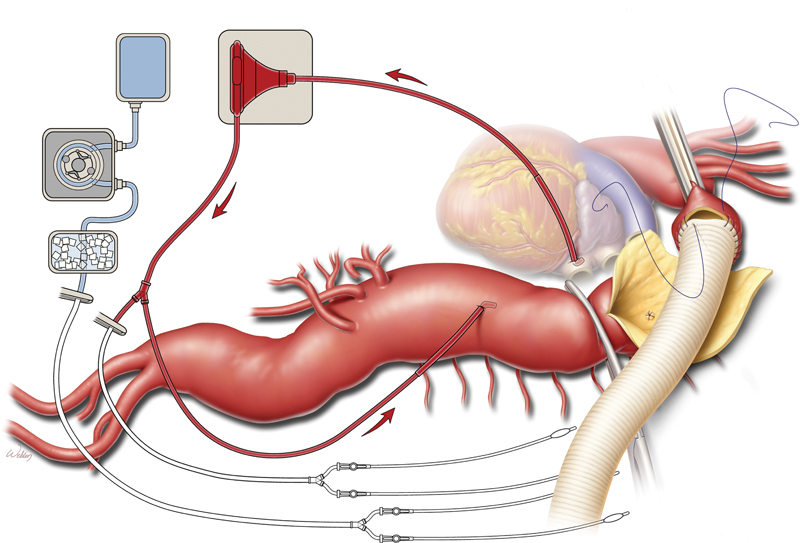
Illustration of a Crawford extent II repair of a thoracoabdominal aortic aneurysm. Left heart bypass is performed while the proximal anastomosis is constructed. The aortic cross-clamp is applied proximal to the left subclavian artery and then moved down the graft after anastomosis is complete. Used with permission of Baylor College of Medicine.

Cooley and DeBakey both used LHB during surgery in the 1950s. Today, we continue to use LHB in more extensive TAAA repairs, cannulating the left atrium and the distal aorta above the celiac axis and then perfusing distally with a centrifugal closed-circuit pump. We retrospectively analyzed a series of 330 extent II TAAA repairs performed from 1986 to 1998. The use of LHB in these extent II procedures ( Fig. 5 ) significantly reduced the incidence of paraplegia. 27 This was a very different outcome from the results obtained when LHB was used in DTA aneurysm replacement. Fig. 5 depicts the LHB in place, with the proximal anastomosis separating the aorta from the esophagus. An appropriately sized graft is selected, and the LHB circuit is later rerouted to perfuse the celiac axis and SMA; cold perfusion to the kidneys is provided by a separate circuit. In our analysis of 3,309 TAAA repairs performed by our group between 1986 and 2014, the permanent paraplegia rate was 2.9% ( *n* = 97). 28 Thus, the risk of postoperative paraplegia has greatly diminished since the Crawford era—probably because of the routine use of CSF drainage and the enhanced perfusion of the distal aorta during repair that is provided during LHB.

In 2002 and 2009, we published our data regarding two randomized clinical trials centered around using cold renal perfusion to protect the kidneys during thoracoabdominal aneurysm repair. 29 30 We discovered that cooling the kidneys significantly improves postoperative renal function. We insert 9-Fr Pruitt balloon catheters into the renal arteries to cool the kidneys.

Just as TEVAR has largely replaced open DTA aneurysm repair, there are emerging endovascular strategies to treat the transverse aortic arch and the thoracoabdominal aorta. There is no doubt that the technology for performing endovascular and minimally invasive percutaneous repairs on virtually every part of the aorta will continue to improve and that these less invasive techniques will eventually dominate aortic surgery. We have progressed from aneurysm ligation to placing homografts to using Dacron grafts to endovascular repairs, but as physicians, we should not stop maintaining our open-surgery skills. We must be cautious about how our practice evolves in the face of emerging technology. Bottom line: aortic surgery will always be an evolution and not a revolution.
